# American Ginseng Extract (*Panax quinquefolius* L.) Is Safe in Long-Term Use in Type 2 Diabetic Patients

**DOI:** 10.1155/2014/969168

**Published:** 2014-05-07

**Authors:** Iva Mucalo, Elena Jovanovski, Vladimir Vuksan, Velimir Božikov, Željko Romić, Dario Rahelić

**Affiliations:** ^1^Centre for Applied Pharmacy, Faculty of Pharmacy and Biochemistry, University of Zagreb, 10 000 Zagreb, Croatia; ^2^Clinical Nutrition and Risk Factor Modification Centre, St. Michael's Hospital, Li Ka Shing Knowledge Institute, Toronto, ON, Canada M5C 2T2; ^3^Departments of Nutritional Sciences and Medicine, Faculty of Medicine, University of Toronto, Toronto, ON, Canada M5S 3E2; ^4^Department of Endocrinology, Diabetes and Metabolic Disease, Dubrava University Hospital, University of Zagreb, 10 000 Zagreb, Croatia; ^5^Clinical Department of Laboratory Diagnostics, Dubrava University Hospital, University of Zagreb, 10 000 Zagreb, Croatia

## Abstract

*Aim*. The objective of the present study was to test the safety of supplementation with the American ginseng (AG) interventional material as an adjunct to conventional therapy (diet and/or medications) in type 2 diabetes, using a double-blind, randomized, placebo-controlled, parallel design. *Methods*. Each participant received either AG (10% ginsenosides) or placebo capsules (500 mg/meal = 3 g/day) for a period of 12 weeks. Outcomes included measures of safety including kidney function (urates and creatinine), liver function (AST and ALT), and haemostatic function (PV and INR). *Results*. Seventy-four participants with well-controlled type 2 diabetes (sex: 28 M and 46 F, age: 63 ± 9.5, BMI: 32 ± 5, and HbA1c: 7 ± 1.3), randomized to either intervention (*n* = 35) or control (*n* = 39) group, completed the study. There was no change in any of the measures of safety between treatments from baseline. The number or severity of adverse events did not differ between the AG intervention and placebo. *Conclusion*. Following 12 weeks of supplementation with AG, safety was not compromised in a high cardiovascular disease (CVD) risk population of patients with type 2 diabetes. This demonstrated that safety is noteworthy, as reviews have continuously warned of possible adverse effects of ginseng consumption.

## 1. Introduction

Despite recent advances in pharmotherapy and disese management, diabetes mellitus continues to be an important public health concern, affecting ~382 million persons in the world [1]. Glycemic management in type 2 diabetes mellitus (T2DM) has become increasingly complex with a widening array of hypoglycemic agents available used as polypharmacy, mounting concerns about their potential interaction and associated adverse effects [2]. At the same time, the care of patients with T2DM has become influenced by a growing interest in complementary and alternative medicine (CAM) including medicinal herbs [3–6]. One of the most promissing therpeutic herbals seems to be the American ginseng.

According to Barnes et al., 14.1% of US adults who used natural health products (NHP) reported ginseng use, making it the fifth most commonly used herbal supplement [7]. This is significant given that 17.7% of the US population uses some form of NHPs for disease prevention or treatment (7). Besides efficacy, in order to obtain a balanced assessment of the clinical application potential of ginseng, it is crucial to consider its safety profile. The available data suggest that ginseng is well tolerated by most users, with the most frequently experienced adverse effects being mild and reversible [6, 8]. Moreover, various animal studies and randomized controlled trials showed that* Panax ginseng* (Asian ginseng) and* Panax quinquefolius* (American ginseng), the two most commonly consumed species of ginseng, exert a neutral or lowering effect on blood pressure [9, 10], thus contrasting Siegel's finding since 1979 [11].

It has previously been stated that until solid evidence is available which demonstrates the safety of specific alternative medicine interventions uncritical acceptance of untested and unproven alternative medicine therapies should proceed with caution [12]. Therefore, to address the paucity of randomized clinical studies assessing ginseng on long-term outcomes in T2DM, we assessed the safety parameters of 12 weeks of supplementation with the American ginseng that in the same study demonstrated positive health benefits, taken in the context of its use adjunct to usual therapy in people with T2DM.

## 2. Patients and Methods

Presented data represents a secondary subset analysis of trial data evaluating effects of AG on glycemic outcome measures (unpublished to date) and vascular outcome measures [13]. Seventy-four participants (35 interventions; 39 placebos) with well-controlled T2DM were recruited from the diabetes outpatient clinic. Inclusion criteria encompassed well-controlled T2DM for >6 months without manifest complications and metabolically stable patients (average HbA1c between 6.5% and 8.1%) on diet and/or conventional diabetes therapies. In addition to diagnosed type 2 diabetes, eligibility criteria included age over 40 years. Exclusion criteria included systolic blood pressure (BP) >160 mm Hg or diastolic BP > 100 mm Hg, secondary hypertension, pregnancy, kidney or liver disease, unstable angina, use of ginseng within 2 months prior to the initiation of the study, and a weight fluctuation of ±2 kg during the intervention periods. All subjects gave informed written consent before taking part in the study, approved by the institutional ethics board (REB number 10/2008). Research followed guidelines of the Declaration of Helsinki and Tokyo.

Participants were randomly assigned to one of the two interventions and received, prior to each of the main meals, three times daily, two 500 mg capsules (total 3 g/day), of either American ginseng extract or identical-appearing placebo capsules containing corn starch. American ginseng root was supplied by the Ontario Ginseng Growers (Simco, ON, Canada), combining five batches from five major farms (ratio 1 : 1 : 1 : 1 : 1) to be representative of entire growing area. The AG intervention was prepared using a conventional ethanol extraction containing 10% total ginsenosides. The dose has been selected based on data from the previously conducted long-term study and derived from the acute-to-chronic clinical testing program for AG [14]. Both interventions were taken simultaneously with usual antihypertensive and hypoglycaemic medications, which included maintenance of the type and dose of such therapies in addition to adherence to dietary recommendations. Randomization to intervention was done using a computer-generated random number table. Subjects, investigators, and statistician were blinded to the identity of the placebo and ginseng capsules by coding and by the indiscernible nature of the capsules.

The study used a randomized, placebo-controlled, double-blind design. The first study phase was a recruitment phase during which interested patients were invited to the clinic to attend an information session providing details about the study procedure. Prospective patients were invited back to the clinic after a 10–12 h overnight fast for screening that included a blood sample and completion of medical questionnaires. Patients who satisfied inclusion criteria proceeded to the second phase where they were randomized to either AG or placebo arm for the 12-week follow-up period. Throughout the intervention phase, patients attended the clinic every 6 weeks (weeks 0, 6, and 12) to have biochemical and anthropometric measurements taken, complete IQOLA SF-36v2 questionnaire [15], receive a new treatment batch, return unused pills, and conduct an interview with the dietician. Patients were advised to maintain initial body weight and follow consistent dietary and physical activity patterns throughout the study. They were also asked to refrain from all medications including AG or placebo during the preceding 12 hours prior to the study visit.

Safety was the outcome measure which included markers of hepatic (aspartate aminotransferase (AST) and alanine aminotransferase (ALT)), renal (serum urates and serum creatinine), and haemostatic (prothrombin time (PT) and international normalized ratio (INR)) functions. Our research group previously found that systolic blood pressure was significantly improved during AG compared with placebo (*P* < 0.001), whereas no significant between-treatment end difference in diastolic BP (DBP) was observed [13]. Finally, body weight change from week 0 to week 12 was not significantly different between AG and placebo and the proportion of pills consumed over the 12 weeks did not differ between the two groups [13].

Samples for hepatic and renal functions, as well as plasma samples for haemostatic function, were analysed at Clinical Department for Laboratory Diagnostics, University Hospital Dubrava. Prothrombin time and international normalized ratio were analyzed on BCS XP analyzer (Siemens, Marburg, Germany). Urea, creatinine, ALT, and AST were determined using standard methods on AU 2700 plus analyser (Beckman Coulter, Tokyo, Japan).

Per-protocol analyses were conducted. Changes in hepatic, renal, and haemostatic functions, were calculated at baseline and after 12 weeks for each intervention. The demographic findings and participant characteristics were compared using a *t*-test for independent samples and *λ* ^2^ test for the intervention and control groups. Comparison between groups and treatment end differences in safety outcome measures is assessed using Factorial ANOVA and Fisher LSD test. Results were expressed as number of participants (*n*), mean ± SD, range (minimum–maximum), and significant at *P* < 0.05. Statistical analyses were performed using STATISTICA v 6.1 (StatSoft Inc., USA).

## 3. Results

**Figure 1:**
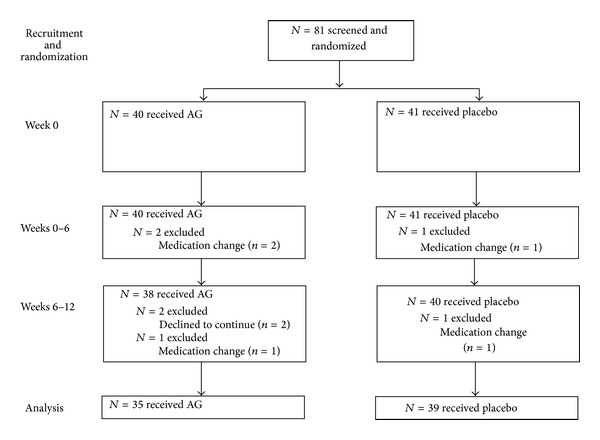
Schematic diagram of the flow of participants through the trial exploring the effect of AG treatment on safety in type 2 diabetic patients.

Eighty-one participants were assessed for their eligibility and enrolled in the study. Their flow through the study is depicted in Figure 1. Dropouts on AG and placebo arms were 5 and 2, respectively. Reasons for dropouts during the protocol included change in medication therapy (*n* = 5) and inability to continue (*n* = 2). Medication change refers to any change in type or dosage of the antihypertensive and/or antihyperglycemic therapy that the patients were receiving. Two patients declined to continue for the following reasons; one was hospitalized due to glaucoma and the other stated lack of time for completion of the study.

Seventy-four participants (28 men and 46 women), aged 63 ± 9.5 years (mean ± SD), with type 2 diabetes, who met the eligibility criteria and were metabolically stable (average HbA1c: 7.1 ± 1.3% and average FPG: 8.3 ± 2.3 mmol/L), completed the study. Usual diabetes treatment received concomitantly by the participants was diet only (*n* = 9) or diet plus oral agents (metformin, *n* = 48; sulfonylurea, *n* = 43; dipeptidyl peptidase-4 inhibitors, *n* = 11; glucagon-like peptide-1 agonist, *n* = 1; acarbose, *n* = 4; metformin + pioglitazone, *n* = 1). Analysis of baseline parameters revealed that the two groups were comparable in all demographic and clinical parameters. However, exception was a baseline body mass index (BMI) and fasting plasma glucose (FPG) which were significantly higher in the group randomized to ginseng arm (Table 1). The proportion of pills consumed over the 12 weeks with compliance above 80% did not differ between the two groups [13].

Within- and between-treatment differences in safety parameters for hepatic, renal, and haemostatic functions were assessed and adverse effects were monitored for AG and placebo. There was no significant dependent or independent effect of treatment and time on safety parameter changes, namely, hepatic function (AST (*P* = 0.107); ALT (*P* = 0.763)), renal function (urates (*P* = 0.498); creatinine (*P* = 0.808)), and haemostatic function (PV (*P* = 0.469); INR (*P* = 0.413)) (Tables 2 and 3). All values were within the normal ranges specified by Clinical Department for Laboratory Diagnostics, University Hospital Dubrava. Furthermore, only one adverse event was reported during the trial. One participant reported stomach heaviness during the first six weeks in the AG group; however, he did not discontinue the AG intervention. The mean absolute AST, ALT, creatinine, urates, PV, and INR values did not differ significantly between both interventions at baseline. There was no effect of study period on safety parameters for hepatic, renal, and haemostatic functions.

## 4. Discussion

The present study represents well-controlled, randomized, clinical study which examined the long-term safety of a ginseng source. Twelve weeks of supplementation with the selected AG extract, dose (3 g/day), and mode of administration (preprandial oral agent taken at −40 min) as an adjunct to usual care in participants with type 2 diabetes demonstrated clinical safety, as assessed by hepatic, renal, and haemostatic functions. None of the outcome safety variables were altered on the AG intervention compared with placebo or baseline values.

Furthermore, the number or severity of adverse events was not significantly different between the AG treatment and placebo; only one adverse event was reported in the intervention group. This demonstrated that safety is noteworthy as the potential for ginseng to cause adverse events and interact with drugs has been debated for the past few decades [16, 17].

The whole safety paradigm around ginseng arouse following an observational study since 1979 where ginseng intake in an observational study was associated with elevated BP and several other adverse effects including gastrointestinal disturbances, insomnia, and nervousness [11]. The adverse effects were observed when taken at doses much higer than the recommended dose, up to 15 g per day where the recommended daily dose is 0.5–2 g. The validity of these side effects is questionable due to a lack of a control group in the study and the fact that subjects were not controlled for dose, duration, route of administration, type of ginseng, or other concurrent bioactive substances intake (e.g., caffeine). When the dose was decreased to 1.7 g/day, the symptoms were rare. Thus the only conclusion that can be validly extracted from the Siegel study is that the excessive and uncontrolled intake of ginseng products should be avoided [18].

Isolated case reports have suggested that* Panax ginseng* (Asian ginseng) was associated with adverse effects ranging from insomnia, diarrhoea, vaginal bleeding, and mastalgia to severe headache, schizophrenia, and the Stevens-Johnson syndrome [19, 20]. Although the exact incidence of these adverse effects is unknown, it seems to be low and related to individual case reports. Complex terminology and lack of clear distinction between various ginseng species, namely, Asian (Chinese or Korean) ginseng (*Panax ginseng*), American ginseng (*Panax quinquefolius*), and Siberian or Russian ginseng (*Eleutherococcus senticosus*) have generated confusion, and it needs to be emphasized that most of the reported adverse effects pertain to* Panax ginseng*.

In 2002, Coon and Ernst systematically reviewed 146 clinical trials representing >8500 individual exposures to ginseng and found it having the same adverse event profile as placebo [8]. The most common events included headache, nausea, restlessness, and sleeplessness which were transient in nature. Ginseng use was found to be well tolerated and its effects were found to be mild and reversible. Furthermore, according to the World Health Organisation (WHO) monograph on selected medicinal plants, American ginseng root has no contraindications and serious adverse effects associated with its use [21].

We administered a dose of 3 g, which is the recommended dose of ginseng in traditional Chinese medicine [22], in alignment with a dose recommended by the Commission E monograph and WHO ginseng monograph [23, 24] and the average intake reported in the study by Siegel. As well, the 12-week treatment period represented the time span in which hypertension developed in Siegel's report. Previously, we found that AG intake at 3 g/day for 12 weeks relative to placebo was associated with a decreasing effect on BP in hypertensive and type 2 diabetic individuals [13]. Stavro et al. reported that hypertensive individuals who choose to consume North American ginseng should be aware of its overall safety and its neutral effect on BP [9].

Several caveats limit the study interpretation. First, the results may not be generalizable to other sources of AG, including the unprocessed root or any other ginseng extracts. It was recently demonstrated that the ginsenoside composition is highly variable [25] and that similar experimentally simulated variability may contribute to equally high variability in its efficacy and safety across batches [26]. This is compounded by the absence of efficacy and safety based standardization. Finally, the absence of follow-up data in the dropouts precluded intention-to-treat analyses to further support the interpretation of the data.

## 5. Conclusion

In conclusion, the selected AG treatment generated rather convincing long-term clinical safety when administered as an adjunct to conventional antihypertensive and antidiabetic therapy. The present study demonstrates that AG did not alter any of the studied safety parameters, namely, renal, hepatic, or haemostatic function. This answers the call posed by the medical establishment for more randomized placebo-controlled clinical studies and provides evidence that ginseng does not cause any more adverse events than placebo. Further long-term studies with different types of ginseng and larger sample size are needed before recommendations are made with regard to its safety.

## Figures and Tables

**Table 1:** Baseline demographic and clinical characteristics of study subjects.

Variables	Group		*P*
	Intervention	Control	
Sample size (*n*)	35	39	
Gender (male/female)	13/22	15/24	0.907
Age (years)	61.9 ± 8.59	63.7 ± 10.28	0.447
Weight (kg)	88.1 ± 17.22	80.9 ± 15.91	0.071
BMI (kg/m^2^)	33.0 ± 5.53	30.1 ± 4.68	0.017
WHR (male)	0.97 ± 0.052	1.00 ± 0.048	0.282
WHR (female) (%)	0.91 ± 0.059	0.97 ± 0.065	0.052
HbA1c (%)	7.2 ± 1.31	6.9 ± 1.21	0.466
FPG (mmol/L)	8.8 ± 2.64	7.7 ± 1.97	0.043
Antihypertensive agents	35	39	0.076
Number taking 1 agent/≥2 agents	20/15	21/18	
Number taking specific agents	BB (8), CCB (9), ACE (16), ARB (0), TD (1), *α*-B (1), mox (2), fixed comb. (19)^#^	BB (5), CCB (9), ACE (24), ARB (2), TD (3), *α*-B (1), mox (2), fixed comb. (17)^#^	
Oral hypoglycemic agents	35	30	0.326
Number taking 1 agent/≥2 agents	10/25	16/14	
Number taking specific agents	MET (25), SULF (27), DPP-4 inh (6), GLP-1 (1), MET + TZD (1), ACA (3)	MET (23), SULF (16), DPP-4 inh (5), GLP-1 (0), MET + TZD (0), ACA (1)	
Hypolipemic agents	27	34	0.707
Number taking 1 agent/≥2 agents	22/5	30/4	
Number taking specific agents	STAT (24), FIB (7), EZE (0), fixed comb. (1)^##^	STAT (32), FIB (4), EZE (1), fixed comb. (1)^##^	

Data expressed as mean ± SD.

BMI: body mass index; WHR: waist-to-hip ratio; HbA1c: glycated hemoglobin; FPG: fasting plasma glucose.

ACE indicates angiotensin-converting enzyme inhibitor; ARB: angiotensin receptor blocker; BB: *β*-blocker; CCB: calcium channel blocker; TD: thiazide diuretic; *α*-B: alpha-blocker; mox: moxonidin (central alpha adrenergic agonist); MET: metformin; SULF: sulfonylurea; DPP-4 inh: dipeptidyl peptidase-4 inhibitor; GLP-1: glucagon-like peptide-1 agonist; ACA: acarbose; TZD: thiazolidinediones; STAT: statin; FIB: fibrate; EZE: ezetemibe; fixed comb.: fixed-dose combination antihypertensives.

^#^ACE + D; ARB + D; ACE + CCB.

^##^STAT + EZE.

*P* value by the independent *t*-test or the Yates corrected chi-square test, as appropriate.

**Table 2:** Change in AST, ALT, creatinine, urates, PT, and INR within control and AG intervention groups.

Parameter	Control group (*n* = 39)		Δ (%)	Intervention group (*n* = 35)		Δ (%)
	Week 0	Week 12		Week 0	Week 12	
AST (U/L)	21.1 ± 7.46	20.9 ± 7.02	0.26 (1.23)	19.6 ± 6.73	18.3 ± 4.17	1.32 (6.74)
ALT (U/L)	24.7 ± 10.95	24.7 ± 11.66	−0.04 (−0.16)	25.5 ± 12.67	23.9 ± 10.25	1.65 (6.47)
Creatinine (*μ*mol/L)	83.78 ± 18.97	85.0 ± 15.47	−1.28 (−1.49)	82.3 ± 15.85	84.1 ± 15.49	−1.74 (−2.11)
Urates (*μ*mol/L)	309.7 ± 68.21	294.8 ± 79.26	14.9 (4.8)	314.1 ± 82.27	311.9 ± 87.49	2.2 (0.7)
PT	100.3 ± 11.53	101.2 ± 10.78	−0.93 (−0.93)	98.1 ± 8.54	98.7 ± 9.35	−0.58 (−0.59)
INR	0.9 ± 0.08	1.0 ± 0.07	−0.01 (−1.01)	1.0 ± 0.08	0.9 ± 0.05	0.04 (3.96)

Data expressed as mean ± SD.

**Table 3:** Between- and within-treatment change from baseline differences in AST, ALT, creatinine, urates, PT, and INR.

Parameter	Control group Week 0 versus week 12^#^	Intervention group Week 0 versus week 12^#^	Week 0 Control group versus intervention group^#^	Week 12 Control group versus intervention group^#^
AST (U/L)	0.868	0.409	0.331	0.107
ALT (U/L)	0.989	0.555	0.760	0.763
Creatinine (*μ*mol/L)	0.753	0.665	0.726	0.808
Urates (*μ*mol/L)	0.596	0.913	0.853	0.498
PV	0.824	0.804	0.523	0.469
INR	0.918	0.168	0.520	0.413

^#^Fisher LSD.
